# Role of Transforming Growth Factor-β1 in Regulating Fetal-Maternal Immune Tolerance in Normal and Pathological Pregnancy

**DOI:** 10.3389/fimmu.2021.689181

**Published:** 2021-08-31

**Authors:** Dongyong Yang, Fangfang Dai, Mengqin Yuan, Yajing Zheng, Shiyi Liu, Zhimin Deng, Wei Tan, Liping Chen, Qianjie Zhang, Xiaomiao Zhao, Yanxiang Cheng

**Affiliations:** ^1^Department of Obstetrics and Gynecology, Renmin Hospital of Wuhan University, Wuhan, China; ^2^Department of Obstetrics and Gynecology, Sun Yat-sen Memorial Hospital, Sun Yat-sen University, Guangzhou, China

**Keywords:** transforming growth factor-β1, pregnancy, immune tolerance, recurrent spontaneous abortion, preeclampsia

## Abstract

Transforming growth factor-β (TGF-β) is composed of three isoforms, TGF-β1, TGF-β2, and TGF-β3. TGF-β1 is a cytokine with multiple biological functions that has been studied extensively. It plays an important role in regulating the differentiation of immune cells and maintaining immune cell functions and immune homeostasis. Pregnancy is a carefully regulated process. Controlled invasion of trophoblasts, precise coordination of immune cells and cytokines, and crosstalk between trophoblasts and immune cells play vital roles in the establishment and maintenance of normal pregnancy. In this systematic review, we summarize the role of TGF-β1 in regulating fetal-maternal immune tolerance in healthy and pathological pregnancies. During healthy pregnancy, TGF-β1 induces the production of regulatory T cells (Tregs), maintains the immunosuppressive function of Tregs, mediates the balance of M1/M2 macrophages, and regulates the function of NK cells, thus participating in maintaining fetal-maternal immune tolerance. In addition, some studies have shown that TGF-β1 is dysregulated in patients with recurrent spontaneous abortion or preeclampsia. TGF-β1 may play a role in the occurrence and development of these diseases and may be a potential target for the treatment of these diseases.

## Introduction

Transforming growth factor-β (TGF-β), an evolutionarily conserved secreted protein consisting of three isoforms, TGF-β1 (the most common), TGF-β2, and TGF-β3, which map to regions of human chromosomes 19q13.1-q13.3, 1q41 (1), and 14q23-24 (2), respectively (3). The role of TGF-β in cell growth, proliferation, differentiation, metabolism, and apoptosis has gradually attracted attention since 1980 (4, 5). Pro-TGF-β1 monomers are composed of a 249-residue domain at the amino terminus, a pro-protein convertase cleavage site, and a 112-residue domain at the carboxyl terminus. TGF-β1 usually exists in the form of latent TGF-β1. Latent TGF-β1 combines with latent TGF-β binding proteins (LTBPs) or glycoprotein-A repetitions predominant protein (GARP) to form large latent complexes (6, 7). Increasing evidence shows that TGF-β1 plays an indispensable role in regulating immune cell differentiation, maintaining immune cell function, and immune homeostasis (8–11).

Healthy pregnancy is a process requiring precise regulation that depends on the balance between the invasion of trophoblast cells and fetal-maternal immune tolerance. The precise coordination and action of various immune cells and cytokines is the key to maintaining fetal-maternal immune tolerance (12). Studies have shown that TGF-β1 plays an important role in trophoblast cell invasion, maintenance of fetal-maternal immune tolerance, and uterine spiral artery remodeling (13–15). In addition, as a multifunctional cytokine, TGF-β1 is widely involved in the regulation of immune cell function and plays an indispensable role in fetal-maternal immune tolerance (16). Therefore, in this review, we specifically focus on the mechanism of TGF-β1 in the fetal-maternal immune tolerance. In addition, we discuss its potential role in the occurrence and development of recurrent spontaneous abortion (RSA) and preeclampsia (PE).

## TGF-β1

### TGF-β1 Activation

TGF-β1 is produced in the form of a precursor. The precursor undergoes processing, such as signal peptide removal, homodimerization, and proprotein convertase cleavage, to produce carboxy-terminal dimers (mature TGF-β1) and amino-terminal dimers (latency-associated peptide, LAP). Mature TGF-β1 and LAP combine in a noncovalent form to form latent TGF-β1 (17). Both immune cells and nonimmune cells can secrete latent TGF-β1. There are two main mechanisms for the extracellular fixation of latent TGF-β1. In the first, TGF-β1 binds to extracellular matrix (ECM) proteins and is deposited in the ECM, which mainly occurs in fibroblasts and epithelial cells. In the second, latent TGF-β1 covalently binds to the transmembrane leucine-rich repeat protein GARP and is maintained on the cell surface, a process that mainly occurs in regulatory T cells (Tregs) (18).

Latent TGF-β1 is inactive because LAP prevents mature TGF-β1 from binding to receptors. The process of releasing mature TGF-β1 from latent TGF-β1 is called TGF-β1 activation (19). The TGF-β1 activation mechanisms include integrin-mediated LAP deformation and release of mature TGF-β1, proteolysis, physicochemical factors, and deglycosylation. These mechanisms have been well summarized in a previous review (20). Here, we emphasize the TGF-β1 activation effect of pregnancy-specific glycoproteins (PSGs). PSGs are encoded by the *Psg* gene on chromosome 19 and are expressed by syncytiotrophoblast cells throughout human pregnancy. There are 10 kinds of PSGs in humans, namely, PSG1-PSG9 and PSG11. PSG1 and PSG9 have previously been confirmed to activate latent TGF-β1, and PSG1 can also inhibit dextran sodium sulfate-induced colitis in mice in a TGF-β-dependent manner (21, 22). It is worth noting that in subsequent studies, researchers have found that all 10 human PSGs can activate TGF-β1, and mouse PSG23 can also activate TGF-β1 in a dose-dependent manner (23). These findings indicate that PSGs are important activators of TGF-β1, and PSGs may play important roles in maintaining immune tolerance during pregnancy by activating TGF-β1.

### TGF-β1 Signaling Pathway

**Figure 1:**
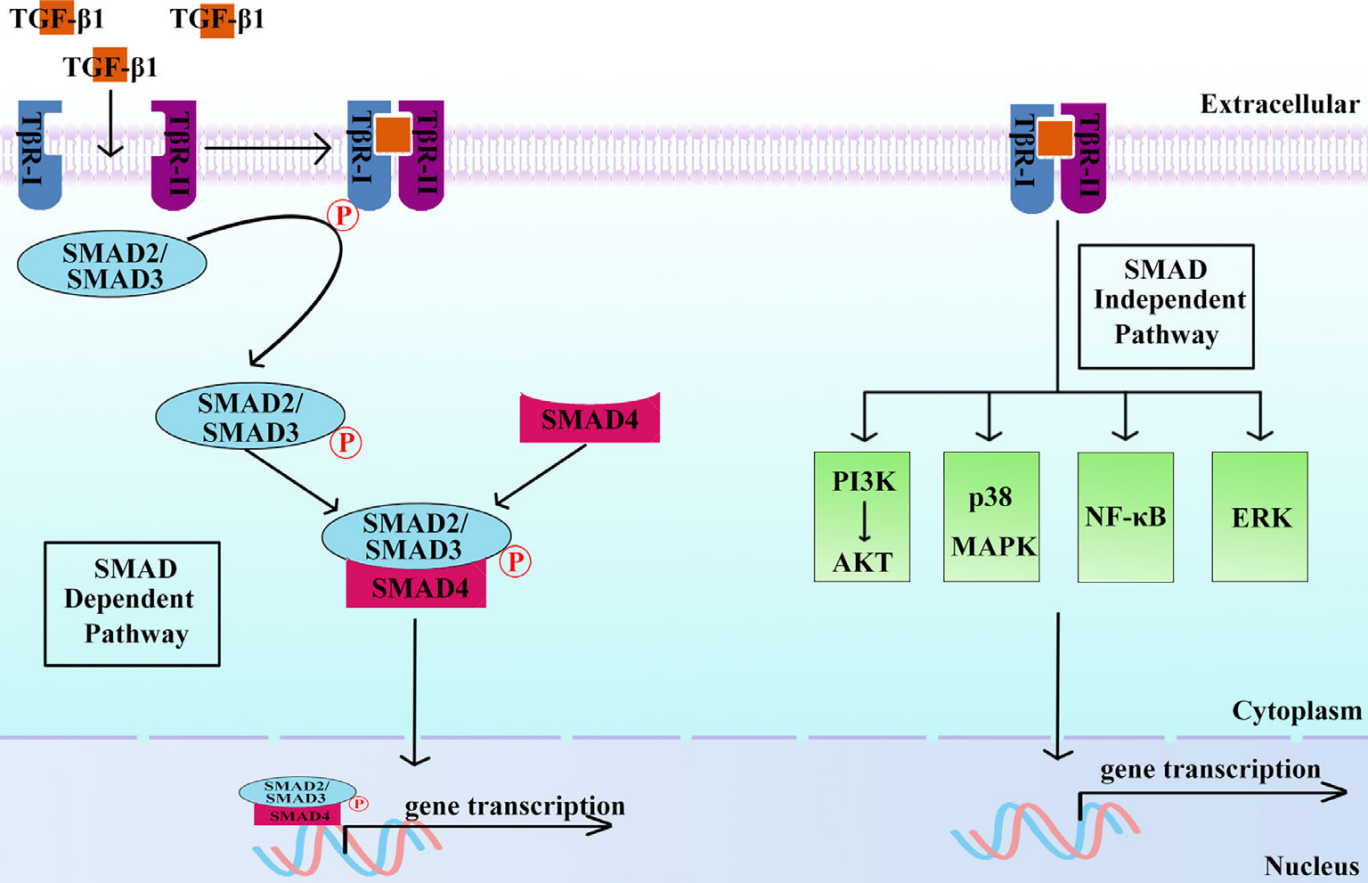
Active TGF-β1 functions *via* SMAD-dependent and/or SMAD-independent pathways. SMAD-dependent pathway: After TGF-β1 binds to specific receptors on the cell surface, phosphorylated TβR-I recruits and phosphorylates SMAD2 and SMAD3. Phosphorylated SMAD2 and/or SMAD3 combine with SMAD4 to form a trimeric complex, which can regulate gene expression in the nucleus. SMAD-independent pathway: After TGF-β1 binds to receptors, phosphorylated TβR-I recruits and phosphorylates signaling molecules, such as PI3K-AKT, p38 MAPK, NF-κB, and ERK, and participates in the regulation of gene expression.

Once activated, mature TGF-β1 binds to high-affinity cell surface-specific receptors and activates downstream signaling pathways to perform corresponding biological functions. Activated extracellular TGF-β1 binds with the transmembrane kinase receptor TGF-β type I receptor (TβR-I) and TGF-β type II receptor (TβR-II) at the cell surface to form a heterotetrameric complex, leading to the phosphorylation and activation of the receptors (24). Subsequently, receptor kinases phosphorylate and activate intracellular cascade signals, including the classic small mother against decapentaplegic (SMAD)-dependent and SMAD-independent pathways (**Figure 1**), and then mediate diverse biological effects (3, 25, 26). SMADs are composed of spherical *N*-terminal DNA-binding domains (MHIs) and *C*-terminal domains (MHIIs), including receptor-regulated SMADs (SMAD1, SMAD2, SMAD3, SMAD5, and SMAD8), common-partner SMAD (SMAD4), and inhibitory SMADs (SMAD6 and SMAD7) (25, 27). In the SMAD-dependent pathway, activated transmembrane receptors phosphorylate serine (Ser) residues at MHII of SMAD2 and SMAD3. Phosphorylated SMAD2 and/or SMAD3 can form a trimeric complex with SMAD4 and then undergo transfer to the nucleus to regulate gene expression (28, 29). In addition to SMAD-dependent pathways, TGF-β1 can also activate SMAD-independent pathways such as PI3K-AKT, p38 MAPK, NF-κB, and ERK to regulate gene expression and participate in regulating cell functions (30–33).

## TGF-β1 in Fetal-Maternal Immune Tolerance

After fertilization, the embryo attaches to the endometrium, and semiallogeneic fetal extravillous trophoblasts (EVTs) begin to invade the uterine mucosa without being rejected by the maternal immune system (34, 35). Once they have invaded into the decidua, EVTs encounter different maternal cell types, such as decidual macrophages, uterine NK (uNK) cells, and Tregs. The moderate invasion of trophoblasts, precise coordination of immune cells and cytokines, and crosstalk between EVTs and immune cells are essential for the establishment and maintenance of a normal pregnancy (36, 37). Tregs, NK cells, macrophages, and other immune cells play vital roles in maintaining fetal-maternal immune tolerance. These immune cells regulate each other and maintain immune homeostasis by secreting proinflammatory or anti-inflammatory cytokines (38). TGF-β1 is a cytokine that exerts a negative regulatory function. It plays important roles in the differentiation of Tregs, the balance of M1/M2 macrophages, and the regulation of NK cell functions.

### TGF-β1 and Tregs

CD4^+^CD25^+^ Tregs are a key subset of T lymphocytes. Both thymus-derived Tregs and peripherally induced Tregs can act as effective inhibitors of inflammatory immune responses and mediate immune homeostasis (39). Natural Tregs depend on the maturation of the thymus, and TGF-β1 is an important inducer of peripheral Tregs (40). FOXP3 is the most specific Treg marker. It is expressed in the thymus and peripheral Tregs regardless of the mode and state of Treg activation (41). The *FOXP3* gene is highly conserved between humans and mice. Mice lacking *Foxp3* usually die of lethal lymphoproliferative autoimmune syndrome, and humans with *FOXP3* mutations suffer from an autoimmune syndrome called IPEX (42, 43). Continuous expression of FOXP3 is necessary to maintain the suppressive immune function of Tregs. Tregs in *Foxp3* ^-^deficient mice lack immunosuppressive function, and Foxp3 transfection can confer CD4^+^CD25^+^ T cells the ability to inhibit the proliferation of CD4^+^ T cells (44).

During pregnancy, the maternal immune system undergoes systemic changes to accommodate the growth and development of fetuses expressing paternal antigens. This immune tolerance is regulated by the number and immunosuppressive functions of Tregs. The number of Tregs increases rapidly in the first trimester, reaches a peak in the second trimester, and gradually decreases to the prepregnancy level during delivery (39, 45). Studies have shown that the proportion of Tregs in the peripheral blood of pregnant women and mice increases significantly during pregnancy, and the specific recruitment of Tregs from maternal peripheral blood to the fetal-maternal interface makes the proportion of Tregs in the placenta and decidua higher than that in peripheral blood (46). Tregs at the maternal-fetal interface prevent fetal rejection by creating an immune tolerance microenvironment characterized by the expression of IL-10, TGF-β1, and heme oxygenase isoform 1 (HO-1) (47). The level and function of Tregs are related to the occurrence and development of pregnancy-related complications such as RSA and PE. CD25^+^Treg depletion can cause embryo implantation failure in allogeneic mice (48). Compared with normal pregnant women, women with spontaneous abortion have a lower level of CD4^+^CD25^+^Tregs (49). It has been appreciated that immunotherapy with paternal or third-party lymphocytes is an effective method of treatment for unexplained RSA (URSA) (50, 51). After immunotherapy with monocytes from the infant’s father, expression of the transcription factor RORγt in Th17 cells in peripheral blood mononuclear cells of URSA patients decreased, while expression of the Treg-specific transcription factor FOXP3 increased, and secretion of the cytokine TGF-β1 related to Tregs increased (52). These results indicate that the Th17/Treg balance is conducive to normal pregnancy, and TGF-β1 seems to be an important factor in regulating the balance of Tregs/Th17 at the maternal-fetal interface.

Tregs can regulate the immune response and maintain immune tolerance through a cell contact-dependent mechanism and a cell contact-independent mechanism. The cell contact-independent mechanism of Tregs is achieved through the secretion of inhibitory cytokines, and TGF-β1 is essential for the proliferation and inhibitory activity of Tregs (53). The activation of TGF-β1 is necessary to induce the production of CD4^+^FOXP3^+^ Tregs, and Tregs can then secrete TGF-β1 and participate in immune regulation (54, 55). Studies have shown that TGF-β1 can promote the differentiation of initial CD4^+^ T cells into Tregs, and TGF-β1 secreted by Tregs can play a role in maintaining the inhibitory properties of Tregs by binding to its receptors. The use of neutralizing antibodies against TGF-β1 or TGF-β1 expression defects in Tregs will result in the weakening or disappearance of Treg inhibitory activity (56–58). The expression of Foxp3 in *TGF-β* ^-/-^ mouse peripheral blood Treg cells was significantly reduced, and the exogenous addition of TGF-β1 could promote the expression of Foxp3 (59). In the CBA/J × DBA/2 abortion-prone mouse model, adoptive transfer of Tregs enhanced the concentration of TGF-β1 in mouse serum and reduced the abortion rate (60). Similarly, applying recombinant IL-17 (a hallmark cytokine secreted by Th17 cells) to the vaginal fornix of pregnant CBA/J mice mated with BALB/c males significantly increased the abortion rate and reduced TGF-β mRNA and protein levels. Adoptive transfer of pregnancy-induced Tregs from 14-day normal pregnant mice before mating offsets the adverse effects caused by IL-17 (61). These results indicate that TGF-β1 is indispensable for maintaining the function of Tregs. Simultaneously, TGF-β1 plays an important role in mediating the fetal-maternal immune tolerance regulated by Tregs, but the specific mechanism requires further study.

### TGF-β1 and NK Cells

NK cells are important components of the endometrial innate immune system and play an important role in the maintenance of pregnancy. In the initial phase of pregnancy, NK cells are preferentially recruited to the endometrium to play an immunomodulatory role under the effect of chemokines derived from endometrial stromal and trophoblast cells (62). TGF-β1 produced by decidual stromal cells can convert CD56^dim^CD16^+^NK cells into CD56^bright^CD16^-^NK cells to complete the terminal differentiation of NK cells (63, 64). Decidual NK (dNK) cells are mainly of the CD56^bright^CD16^-^phenotype, constituting between 50 and 70% of the total lymphocytes of the decidua, and they are the most abundant immune cells in the decidua (65). Low cytotoxic dNK cells can regulate vascular remodeling at the maternal-fetal interface by producing vascular endothelial growth factor, angiopoietin, and TGF-β1 (66–68). An emerging mechanism by which mesenchymal stem cells (MSCs) regulate the immune function of dNK cells has gradually attracted more attention (69). Studies suggest that menstrual blood stromal/stem cells (MenSCs), a substitute for endometrial MSCs, can induce the proliferation of NK cells. However, MenSCs pretreated with IFN-γ can suppress NK cell proliferation by releasing TGF-β and IL-6 (70). These results indicate that TGF-β1 is an important mediator that regulates the function of dNK cells and plays an important role in the maintenance of fetal-maternal immune tolerance.

### TGF-β1 and Macrophages

Almost 20-30% of the leukocytes in the decidua in the first trimester are macrophages. Macrophages play important roles in trophoblast cell invasion, vascular remodeling, and immune tolerance (71). Macrophages are classified into two subpopulations: classic M1 and alternative M2 macrophages. M1 macrophages express proinflammatory factors such as IL-6, IL-12, and TNF-α. Conversely, M2 macrophages upregulate the expression of anti-inflammatory cytokines such as TGF-β1 and IL-10 (72). The elaborate balance between M1 macrophages and M2 macrophages is of prime importance to establish and maintain pregnancy (73–75). M2 macrophages promote cell homeostasis by secreting TGF-β1 and IL-10, which has a profound impact on maintaining the immune tolerance environment (76).

Macrophages not only regulate local immune function but also directly promote the migration and invasion of extravillous trophoblast cells and support spiral artery remodeling and angiogenesis (77). Coculture of trophoblasts and macrophage cell lines can promote the polarization of macrophages to M2, and expression of the marker proteins TGF-β1 and IL-10 *via* the IL-6/STAT3 pathway and M2 macrophages can promote the invasion and migration of trophoblasts (78). Unlike NK cells, which are only located in the decidua, macrophages exist in both the decidua and the placenta and are the major immune cell population in the placenta. Placental macrophages have also shown an M2-like phenotype; genes related to M1 are silenced by hypermethylation, while genes related to M2 are hypomethylated (79). A recent study has shown that placental macrophages have two cytokine expression patterns. In the first pattern, placental macrophages produce reduced levels of IL-1, IL-6, IL-10, IL-8, and TNFα and can be stimulated by bacterial endotoxins. In the second pattern, placental macrophages constitutively express IL-11, IL-17A, IL-17F, TGF-β1, and VEGF, and this expression is unresponsive to stimulation (80). Taken together, this evidence reveals that TGF-β1 plays an important role in regulating the function of macrophages at the maternal-fetal interface.

### TGF-β1 and Regulatory B Cells

Regulatory B cells (Bregs), a new type of B cell population, have a negative immunomodulatory effect. Bregs are a collective term for a variety of regulatory B cell subgroups, accounting for approximately 0.5% of the total number of B cells in healthy people (81). Similar to Tregs, Bregs can maintain immune tolerance by producing different inhibitory cytokines (such as IL-10, IL-35, and TGF-β1) or through cell contact-dependent mechanisms (82). Bregs can interact with Tregs, macrophages, and dendritic cells to participate in the regulation of immune homeostasis (83). A large number of studies have found that Bregs also play an important role in maintaining fetal-maternal immune tolerance. Studies have shown that increased levels of CD5^+^CD1d^+^Breg cells can reduce immune abortion in pregnant mice. The adoptive transfer of Bregs to abortion-prone mice can enhance the function of Tregs and maintain the immature state of DCs to enhance maternal immune tolerance (84). In addition, estrogen can also induce the maturation of Bregs during pregnancy to maintain immune tolerance (85).

Bregs play a key role in the downregulation of the inflammatory response through an IL-10-dependent mechanism (86). In addition, compared with normal mice, the percentage of Bregs expressing IL-35 in the peripheral blood of abortive mice is reduced, suggesting the potential involvement of IL-35 in pregnancy maintenance (87). Can the TGF-β1 released by Bregs also participate in the regulation of the immune balance of the maternal-fetal interface? At present, research is lacking on Bregs involved in regulating the immune tolerance of the maternal-fetal interface by releasing TGF-β1. Considering that Bregs are an established source of TGF-β1, TGF-β1 can regulate a variety of immune cell functions, including Tregs, and the important role of Bregs during pregnancy, more in-depth research remains to be conducted.

## TGF-β1 in Pathological Pregnancy

The two key events of normal human pregnancy are embryo implantation and placenta formation (88). During these events, the maternal immune system must accept the genetically incompatible fetuses to allow trophoblast invasion (89). It is known that pregnancy-related diseases, such as RSA and PE, may be closely related to impaired immune tolerance (90, 91). TGF-β1 is an important regulator of immune cell function. Understanding the role of TGF-β1 in the occurrence and development of RSA and PE may help to further reveal the etiology of these diseases.

### RSA

RSA refers to two or more consecutive spontaneous abortions before 20 weeks of pregnancy, and its incidence is approximately 5% (92–94). Studies have confirmed that compared with healthy controls, the expression of TGF-β1 in the decidual tissue of RSA patients is significantly decreased (95, 96). Treatment with vasoactive intestinal peptide (VIP) in a miscarriage-prone mouse model (CBA/J×DBA/2) can regulate the endocytosis of maternal macrophages and promote the expression of TGF-β1 at the implantation site of the mouse, significantly increasing the number of implant points (97). In addition, Ma et al. (98) found that endovascular extravillous trophoblasts (enEVTs) actively produced TGF-β1, and primary enEVTs promoted the differentiation of naive CD4^+^ T cells into immunosuppressive Tregs in a TGF-β1-dependent manner. The proportion of TGF-β1-producing enEVTs and their ability to educate Tregs differentiation were significantly reduced in RSA patients. Overall, TGF-β1 may participate in the occurrence and development of RSA by regulating immune tolerance.

The TGF-β1 signaling pathway can regulate fetal-maternal immune tolerance by regulating the expression of indoleamine 2,3-dioxy (IDO), thereby participating in immune and inflammatory responses (99). In semen, high concentrations of TGF-β1 and TGF-β2 can regulate women’s immune tolerance to sperm, embryo implantation, and subsequent pregnancy. After intercourse, the pH of the vagina will activate TGF-β-involved immune tolerance (100). Furthermore, existing studies show that TGF-β seems to be a factor controlling the apoptosis and proliferation of endometrial cells during the process of embryo implantation. Every subtype of TGF-β has a different effect on the endometrium; TGF-β1 and TGF-β2 can induce the apoptosis of uterine cells, while TGF-β3 has a proliferation-promoting effect (26). In summary, in addition to immune cells, TGF-β1 is also involved in maintaining the functions of nonimmune cells.

### PE

PE is a pregnancy-specific disease characterized by new hypertension and proteinuria after 20 weeks of gestation. Its global incidence is approximately 5-8% (101). PE can be divided into two different subtypes: early-onset PE (appearing before 34 weeks) and late-onset PE (appearing after 34 weeks). The pathogenesis between these two subtypes is different (102). Although some theories have been proposed to explain PE, its pathogenesis has not yet been elucidated.

A study found that compared with the control group, the expression of TGF-β1 in maternal and cord blood of late-onset PE was significantly reduced, but there was no significant difference in that of early-onset PE (103). However, most studies have not clearly pointed out whether PE patients have early- or late-onset PE. For example, in a study on the TGF-β1 single nucleotide polymorphism and the risk of PE in Chinese women, the researchers did not indicate whether PE patients were early- or late-onset. PE patients were divided into mild PE and severe PE. It was found that the allelic variant of TGF-β1 rs1800469 T was associated with the risk of PE, and TGF-β1 rs1800469 T>C was negatively correlated with the severity of PE (104). In addition, a study has shown that compared with healthy controls, the expression of TGF-β1 and Smad3 is upregulated in the placenta of patients with PE (105), while another study has shown no significant difference in the expression level of TGF-β1 in the serum of controls and PE patients (106). These contradictory results may be due to the different tissues tested, the racial differences in the subjects studied, and the small number of subjects in some studies. In addition, these studies rarely mentioned whether the detected TGF-β1 was active. It should be noted that due to ethical requirements, most clinical specimens of PE are derived from the placenta obtained after delivery (>34 weeks), which cannot well reflect the early development of PE. Therefore, these research results should be considered with caution. Establishing TGF-β1 testing standards and carrying out large-scale clinical sample testing may be effective solutions to determine whether the expression level of TGF-β1 is related to PE.

Impaired trophoblast cell invasion and increased uterine placental vascular resistance in early pregnancy are important pathological mechanisms of PE (107). TGF-β1 is involved in regulating the invasion of human trophoblast cells (108, 109). Compared with healthy controls, the transcription level of *TGFB1* and the level of active TGF-β1 protein are increased in the placental tissue of patients with PE. The TGF-β1/Smad3 signaling pathway can mediate the inhibition of trophoblast cell migration and invasion caused by the downregulation of lysyl oxidase (LOX) (110). Liu (111) et al. found that TGF-β1/Smad3 can also mediate the inhibitory effect of miR-142-3p on the invasion and migration of trophoblast cells *in vitro*. In addition, TGF-β1 is also involved in the regulation of endothelial cell function. Endoglin is a coreceptor of TGF-β signaling. Soluble endoglin increases significantly in the serum of PE patients, and its level is related to the severity of PE. Soluble endoglin can block the vasodilation induced by TGF-β1 in rats by inhibiting the binding of TGF-β1 to its receptor (112). A study on the underlying mechanism between maternal PE and fetal vascular function found that compared with the offspring of healthy controls, the TGF-β1 signaling network in HUVECs of maternal PE offspring was impaired. Maternal PE can promote the proliferation of female fetal HUVECs induced by TGF-β1, but it has no effect on the proliferation of male fetal HUVECs induced by TGF-β1. These findings suggest that TGF-β1 may be involved in the regulation of endothelial cell function, and maternal PE plays different roles in the regulation of female and male fetal endothelial cell function (113). Although the above studies have not clearly pointed out whether PE is early- or late-onset PE, they indicate that TGF-β1 may participate in the occurrence and development of PE by regulating the invasion of trophoblast cells and the function of vascular endothelial cells.

PE is related to abnormalities of the immune system throughout the body and the placenta. Studies have shown that compared with healthy controls, the level of TGF-β1 in the decidua of PE patients (not indicating early- or late-onset PE) is increased, and high levels of TGF-β1 can inhibit the activation of specific subgroups of dNK cells, thereby participating in the occurrence of PE (114). In addition, a mass spectrometry study found that the level of PSG9 in the serum of women with early-onset PE was significantly higher than that in the control group, and PSG9 may be a potential marker of PE (115). Another study has shown that PSG9 can bind to LAP and activate potential TGF-β1 to induce the production of FoxP3^+^ Tregs, indicating that PSG9 may be involved in inducing immune tolerance at the fetal-maternal interface (21). These results are contradictory, and the role of PSG9 in the pathogenesis of PE remains to be revealed by more in-depth studies. Although these results suggest that TGF-β1 may participate in the occurrence of PE by regulating immune tolerance, further research is needed to explore the role and specific mechanisms of TGF-β1.

## Conclusion

In summary, TGF-β1 plays an important role in regulating the function of immune cells at the maternal-fetal interface and maintaining immune homeostasis. TGF-β1 can induce the production of Tregs, regulate the balance of Tregs/Th17, and is necessary to maintain the suppressive immune function of Tregs. In addition, TGF-β1 can induce the production of CD56^bright^CD16 NK cells, and TGF-β1 released by dNK cells participates in the regulation of vascular remodeling at the maternal-fetal interface. Furthermore, TGF-β1 plays a vital role in regulating the M1/M2 balance. TGF-β1 is a cytokine secreted by Tregs, NK cells, and M2 macrophages. It may be an important molecule that coordinates the balance of immune cells at the maternal-fetal interface and maintains immune tolerance.

During pregnancy, TGF-β1 and TGF-β2 induce endometrial cell apoptosis, while TGF-β3 promotes endometrial cell proliferation. The differential regulation of TGF-β subtypes on endometrial cells may be the key regulatory mechanism of endometrial decidualization. In addition, TGF-β1 is differentially expressed in RSA tissues, and the dysregulation of TGF-β1 may be related to the occurrence and development of RSA. Although the expression level of TGF-β1 in the decidua and serum of patients with PE is still controversial, an increasing number of studies have shown that TGF-β1 can participate in the occurrence and development of PE by affecting the invasion ability of trophoblast cells and the activation of dNK cells. Therefore, TGF-β1 may be a potential therapeutic target for these diseases, and the role of TGF-β1 in the immune tolerance of the maternal-fetal interface may provide new clues for the immunological treatment of pregnancy-related complications.

Because TGF-β1 plays a key role in regulating trophoblast cell invasion and fetal-maternal immune tolerance, the development of specific drugs targeting TGF-β1 for the treatment of pregnancy-related diseases may have broad prospects. However, since TGF-β1 is expressed in almost all cells, strategies to target the drug to the maternal-fetal interface to function face challenges that must be overcome.

Current studies have certain limitations. For example, many studies do not clearly indicate which TGF-β subtype was studied but instead described it as TGF-β, raising confusion and obstacles to summarizing the functions of TGF-β1 and its role in diseases. In addition, most studies on the relationship between TGF-β1 and immune cells were carried out in cell lines. The use of human or mouse primary cells may be more conducive to revealing the true role of TGF-β1 in regulating immune cell function. In future studies, standardizing the expression and detection standards of TGF-β subtypes and conducting more primary cell or *in vivo* studies may further reveal the role of TGF-β1 in pathophysiological conditions.

## Author Contributions

DY, FD, XZ and YC proposed this idea. FD, DY, MY, SL, YZ, WT, ZD, LC and QZ performed the PubMed search. FD and DY drafted the manuscript. XZ and YC edited/reviewed this review. All authors agree to be accountable for the content of the work. All authors contributed to the article and approved the submitted version.

## Funding

This review was supported by the National Key R&D Program of China (grant number 2018YFC1003200), the National Natural Science Foundation of China (grant number 81860276, 82071655), China Medical Association Clinical Medical Research Special Fund Project (grant number 17020310700), the Fundamental Research Funds for the Central Universities (grant number 2042020kf1013), Educational and Teaching Reform Research Project (grant number 413200095), and Graduate credit course projects (grant number 413000206).

## Conflict of Interest

The authors declare that the research was conducted in the absence of any commercial or financial relationships that could be construed as a potential conflict of interest.

## Publisher’s Note

All claims expressed in this article are solely those of the authors and do not necessarily represent those of their affiliated organizations, or those of the publisher, the editors and the reviewers. Any product that may be evaluated in this article, or claim that may be made by its manufacturer, is not guaranteed or endorsed by the publisher.
